# *R2d2* and Hyperdrive Mechanisms (in Mouse Meiosis)

**DOI:** 10.1371/journal.pgen.1004950

**Published:** 2015-02-13

**Authors:** Sarah E. Zanders, Harmit S. Malik

**Affiliations:** 1 Division of Basic Sciences, Fred Hutchinson Cancer Research Center, Seattle, Washington, United States of America; 2 Howard Hughes Medical Institute, Fred Hutchinson Cancer Research Center, Seattle, Washington, United States of America; University of Wisconsin–Madison, UNITED STATES

*“Mendelism is a magnificent invention for fairly testing genes in many combinations, like an elegant factorial experimental design. Yet it is vulnerable at many points and is in constant danger of subversion by cheaters that seem particularly adept at finding such points.”*

- *James F. Crow* [1]

Mendelian transmission is established during meiosis, the cell division that generates haploid gametes (e.g., sperm and eggs) from diploid germ cells. Meiosis does not, however, have to be fair. Selfish genetic elements, or meiotic drivers, have evolved to cheat this process in order to be packaged into functional gametes more often than the expected 50% probability. By biasing allele transmission in their favor, meiotic drive alleles can short-circuit natural selection, causing their spread even if they are harmful to organismal fitness. Indeed, meiotic drive alleles are thought to be directly or indirectly associated with infertility in diverse eukaryotes, from fungi to flies to humans [2]. Drive occurring in male meiosis may have immediate consequences to fertility, potentially facilitating its detection. In contrast, drive in female meiosis could lead to very subtle skews in inheritance patterns and no overt signs of infertility; such driver alleles may therefore go unnoticed even if they are more pervasive [3, 4]. Surprisingly few meiotic drivers have been conclusively identified. There are three major roadblocks to the efficient identification and validation of meiotic drive alleles. First is the prevalent usage of isogenic, lab-domesticated organisms in genetics research; driver alleles can only be identified in heterozygotes. Second, the rapid evolution of suppressors to alleviate the deleterious effects of meiotic drivers can obscure drive in intraspecific crosses and also rapidly extinguish meiotic drive alleles by eliminating their evolutionary advantage [5]. Third, it can often be difficult to distinguish the actions of meiotic drive alleles from inherent viability defects associated with the underrepresented allele. Overcoming many of these limitations, a new report in this issue of *PLOS Genetics* by de Villena and colleagues provides compelling evidence for a massive copy number expansion that is causally linked to female meiotic drive in mice [6].

Didion et al. began their study by investigating the genetic basis of transmission ratio distortion (TRD) of a region of mouse chromosome 2. This TRD had been previously observed in a number of crosses, including, most recently, in the recombinant inbred Collaborative Cross (CC) lines designed for genetic analyses [7]. TRD was observed in favor of the WSB/EiJ allele across a ~50 Mb region in the middle of chromosome 2 (WSB/EiJ was one of the eight original inbred strains used to create recombinant inbred lines [5]). In the present report, Didion et al. first confirmed the TRD favoring the WSB/EiJ allele in the Diversity Outbred (DO) population of outbred mice derived from CC lines designed for mapping traits. Crosses of various WSB/EiJ heterozygotes to tester strains showed that TRD was specific to heterozygous females. These findings already eliminated the possibility of postmeiotic dysfunction of selective male gametes as the basis for TRD; such postmeiotic dysfunction is the basis for TRD caused by Segregation Distorter in *Drosophila melanogaster* [8] and the t-haplotype in mouse [9].

This finding left open two possibilities, the first being that the bias originates from a selective postfertilization defect. Indeed, Didion et al. find that the amount of TRD does negatively correlate with litter size, suggesting that lethality of oocytes or embryos inheriting the non-WSB/EiJ allele could contribute to the TRD. However, the observed decreases in litter sizes are insufficient to explain the magnitude of TRD observed. In addition, despite the decreased litter sizes, the absolute number of progeny inheriting the WSB/EiJ allele in crosses exhibiting TRD was significantly higher than the number inheriting the allele in crosses without TRD. Thus, although embryonic lethality occurs in crosses showing TRD, Didion et al. propose that “true meiotic drive” must occur to fully explain the TRD in favor of the WSB/EiJ allele. Such drive occurs because, in female meiosis, only one of four meiotic products is selected in the oocyte [10].

**Figure 1:**
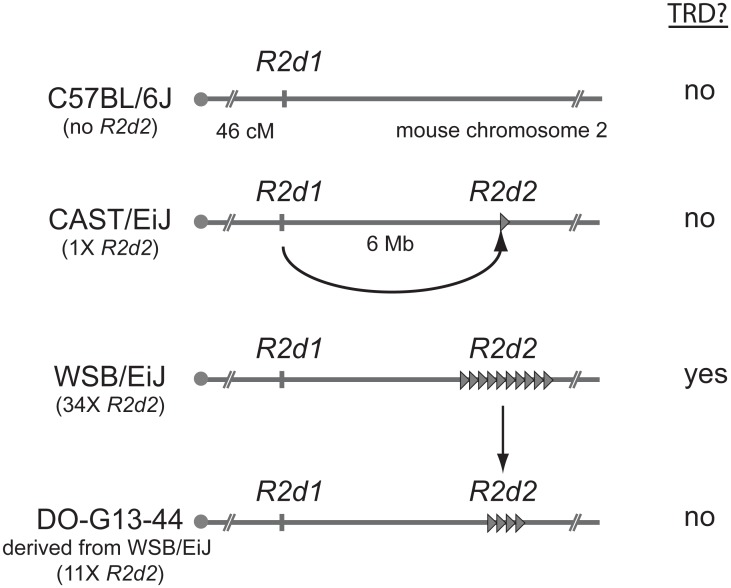
Transmission ratio distortion (TRD) caused by a high copy *R2d2* array. In the absence of the *R2d2* sequence (e.g., C57BL/6J), or when the copy number of the sequence is low (e.g., CAST/EiJ), allele transmission through heterozygous females is Mendelian and no embryonic lethality is observed. When the copy number is high in the appropriate genetic background (e.g. WSB/EiJ), the litter sizes of heterozygous females are reduced and the *R2d2* locus shows TRD. When the copy number of *R2d2* repeats decreases (e.g., DO-G13–44), litter sizes increase and Mendelian segregation is restored.

Based on the meiotic drive inference, the authors dubbed the causative allele *R2d2* (Responder to drive on chr. 2). To map *R2d2*, Didion et al. again exploited the impressive genetic power of the CC and DO lines. By genotyping over 400 mice, they identified recombination events within the candidate region on chromosome 2. All eight recombinant chromosomes that showed TRD shared a 9.3 Mb region containing a 127 kb unit of DNA (*R2d2*) that is repeated ~36 times in WSB/EiJ (see Fig. 1). Three other mice strains previously shown to exhibit TRD also contain *R2d* sequences at a high copy number. In contrast, R2d is only present at 1–2X copy number in the reference mouse genome and other mouse strains that lack the TRD phenotype. In non-TRD strains, like the reference genome, the *R2d* sequences appear to be present only in the (presumed) ancestral location spread over a 158 kb region (R2d1) ~6 Mb distal from the *R2d2* locus (Fig. 1A). These results suggested the hypothesis that whereas all mouse strains contain *R2d1*, a copy number expansion at *R2d2* is causative for TRD (Fig. 1). A satisfying confirmation of the hypothesis emerged from the genetic instability of the *R2d2* repeat cluster. Didion et al. identified a WSB/EiJ-derived female in which one of the *R2d2* repeat arrays had collapsed from ~34 to ~11 copies. This collapse resulted in both the loss of TRD and increased litter sizes (Fig. 1). Together, this beautiful series of experiments, relying on both classical genetics and genomic assembly mapping, identify and confirm the *R2d2* expansion as causal for female meiotic drive.

How does *R2d2* expansion cause meiotic drive? Didion et al. favor the model that the *R2d2* expansion leads to preferential inheritance in meiosis. Such non-Mendelian inheritance would occur via the formation of neocentromere-like activity on cis-acting sequences expanded in the *R2d2* cluster, which favor their preferential orientation and thereby inclusion in the oocyte [11]. This situation would be highly reminiscent of the knob elements in maize that also take advantage of female meiosis asymmetries to increase their likelihood of inclusion into the oocyte [12]. Akin to knob elements, *R2d2* drive might occur in Meiosis II [3, 4]. Under this model, the ensuing *R2d2*-dependent embryonic lethality would result from generating eggs that are aneuploid for chromosome 2.

Not all dams heterozygous for *R2d2* show TRD, implicating the requirement of at least one additional modifier locus that must be present to manifest TRD. These additional loci could represent allelic variants of meiotic drive suppressors, which reduce the harmful fitness effects of drive and associated embryonic lethality. Alternatively, these could represent “trans-acting factors” (distorters) that bind *R2d2* and endow it with microtubule attachment or motor function that results in neocentromere activity [12]. These findings would be at odds with predictions from the theory that distorter and responder loci ought to be tightly linked, so as to not be separated by recombination [13]. One intriguing possibility is that the only protein encoded by the *R2d* unit, Cwc2, might itself bind the *R2d2* cluster and contribute to TRD.

In sum, the paper by Didion et al. represents a tour de force in characterizing a complex multilocus TRD system in a genetically tractable mammalian model system. Uncovering the molecular mechanisms underlying the transmission of exceptional meiotic drive alleles like *R2d2*, as well as their evolutionary origins, will broaden our general understanding of fertility and chromosome segregation. Such work also reinforces previous findings about the insidious and relentless nature of meiotic competition between chromosomes [1, 2, 10].
